# Clinical validation of an immunohistochemistry‐based CanAssist‐Breast test for distant recurrence prediction in hormone receptor‐positive breast cancer patients

**DOI:** 10.1002/cam4.2049

**Published:** 2019-03-07

**Authors:** Manjiri M. Bakre, Charusheila Ramkumar, Arun Kumar Attuluri, Chetana Basavaraj, Chandra Prakash, Ljubomir Buturovic, Lekshmi Madhav, Nirupama Naidu, Prathima R, S. P. Somashekhar, Sudeep Gupta, Dinesh Chandra Doval, Mark D. Pegram

**Affiliations:** ^1^ OncoStem Diagnostics Private Limited Bangalore India; ^2^ Clinical Persona Inc East Palo Alto California; ^3^ Manipal Hospital and Comprehensive Cancer Centre Bangalore India; ^4^ Tata Memorial Hospital Mumbai India; ^5^ Rajiv Gandhi Cancer Institute New Delhi India; ^6^ Stanford University Medical Center Palo Alto California

**Keywords:** CanAssist‐Breast, distant recurrence, early‐stage breast cancer, immunohistochemistry, prognostication, support vector machine

## Abstract

CanAssist‐Breast (CAB) is an immunohistochemistry (IHC)‐based prognostic test for early‐stage Hormone Receptor (HR+)‐positive breast cancer patients. CAB uses a Support Vector Machine (SVM) trained algorithm which utilizes expression levels of five biomarkers (CD44, ABCC4, ABCC11, N‐Cadherin, and Pan‐Cadherin) and three clinical parameters such as tumor size, grade, and node status as inputs to generate a risk score and categorizes patients as low‐ or high‐risk for distant recurrence within 5 years of diagnosis. In this study, we present clinical validation of CAB. CAB was validated using a retrospective cohort of 857 patients. All patients were treated either with endocrine therapy or chemoendocrine therapy. Risk categorization by CAB was analyzed by calculating Distant Metastasis‐Free Survival (DMFS) and recurrence rates using Kaplan‐Meier survival curves. Multivariate analysis was performed to calculate Hazard ratios (HR) for CAB high‐risk vs low‐risk patients. The results showed that Distant Metastasis‐Free Survival (DMFS) was significantly different (*P*‐0.002) between low‐ (DMFS: 95%) and high‐risk (DMFS: 80%) categories in the endocrine therapy treated alone subgroup (n = 195) as well as in the total cohort (n = 857, low‐risk DMFS: 95%, high‐risk DMFS: 84%, *P *<* *0.0001). In addition, the segregation of the risk categories was significant (*P *=* *0.0005) in node‐positive patients, with a difference in DMFS of 12%. In multivariate analysis, CAB risk score was the most significant predictor of distant recurrence with hazard ratio of 3.2048 (*P *<* *0.0001). CAB stratified patients into discrete risk categories with high statistical significance compared to Ki‐67 and IHC4 score‐based stratification. CAB stratified a higher percentage of the cohort (82%) as low‐risk than IHC4 score (41.6%) and could re‐stratify >74% of high Ki‐67 and IHC4 score intermediate‐risk zone patients into low‐risk category. Overall the data suggest that CAB can effectively predict risk of distant recurrence with clear dichotomous high‐ or low‐risk categorization.

## 1 INTRODUCTION

Despite the advent of multigene assay formats for breast cancer prognosis, great disparities exist in under‐resourced jurisdictions globally, with respect to the availability of feasible and affordable tests for early‐stage breast cancer prognosis and treatment planning. Trials have shown that the Hormone‐Receptor (HR)‐positive and HER2/neu (Human Epidermal Growth factor receptor‐2)‐negative early‐stage breast cancer patients have sustained risk of recurrence over a period of 5‐20 years^1^, ^2^ and rates of distant recurrence in patients treated with endocrine therapy alone is 15% in the first 5 years.^1^

Several multigene tests such as Oncotype Dx,^3^ MammaPrint,^4^ Prosigna,^5^ and EndoPredict^6^ have been developed to stratify ER‐positive early‐stage breast cancer patients. The TAILORx prospective trial showed that a total of 85% of patients (low‐ and intermediate‐risk) enrolled in this trial did not benefit from chemotherapy.^7^, ^8^ Results of another prospective trial, MINDACT^9^showed that chemotherapy did not benefit patients who were clinically high‐risk but genomically low‐risk. Notwithstanding the wide utility of the multigene tests, they are not impactful in Asian countries owing to the high cost of the test and the lack of validation data on Asian patient cohort.

Immunohistochemistry (IHC) is a widely used and less expensive methodology as compared to genomics‐based technologies used in the multigene tests. IHC4 score^10^ and PREDICT^11^ are immunohistochemistry‐based tests that use the expression of breast cancer biomarkers‐estrogen receptor (ER), progesterone receptor (PR), Ki‐67, and HER2/neu for prognostication. IHC4 score has demonstrated that its prognostic clinical utility is comparable to that of multigene test, Oncotype Dx.^12^ Ki‐67 expression status alone is used by several physicians to tailor therapy decisions. However, the lack of standardized protocols for IHC performance and grading procedures for Ki‐67^13^ across different laboratories could lead to interlaboratory variations in turn affecting treatment decisions.

A robust statistical model is equally important for a multigene/biomarker‐based test to perform accurately. Regression analysis used in multigene tests has been shown to lack high levels of accuracy.^14^ In a comparative analysis, Support Vector Machine (SVM) model for breast cancer (BCRSVM) outperformed other models like Cox Proportion Hazard regression and Artificial Neural Network (ANN) with high accuracy.^15^

Selection of biomarkers reflective of aggressive tumor biology is integral to the clinical utility of any multigene test. Most markers used in the current multigene tests are involved in cell proliferation. However, there are additional mechanisms like EMT (Epithelial‐Mesenchymal transition), loss of cell‐cell adhesion, MET (Mesenchymal‐Epithelial transition), drug resistance that are known to play a role in metastasis and recurrence and have not been part of current tests.^16^, ^17^

**Figure 1:**
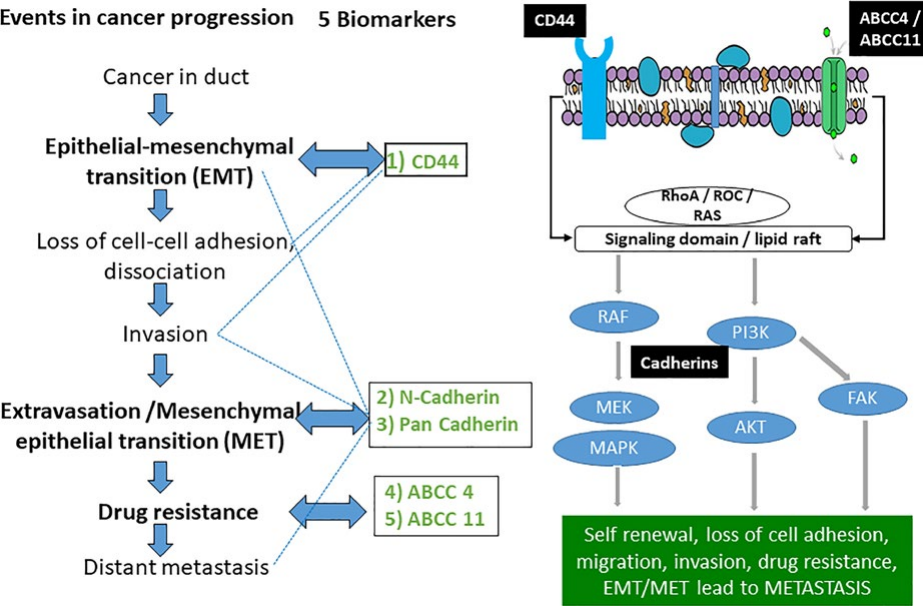
Rationale of biomarker selection: Role and cross talk between the biomarkers chosen for test development during cancer progression. Five selected biomarkers for CanAssist‐Breast (CD44, Pan‐Cadherin, N‐Cadherin, ABCC4, and ABCC11) participate in various steps of cancer progression and are also involved in the cross talk (shown by dotted line)

Keeping the above points in mind, we have developed an immunohistochemistry‐based CanAssist‐Breast (CAB) test^18^ using a SVM‐based model based on three clinical parameters (Tumor size, grade, and node status) plus IHC grading information from the five biomarkers. CAB predicts risk of distant recurrence within 5 years from diagnosis for HR+ and HER2− early‐stage breast cancer patients. Our approach to the biomarker selection for CAB namely CD44, N‐Cadherin, Pan‐Cadherin, ABCC4, and ABCC11 was to focus on diverse signaling pathways that regulate key steps in cancer metastasis and drug resistance (Figure 1).

In this manuscript, we present data on the clinical validation of CanAssist‐Breast using a retrospective cohort of 857 patients. The prognostic utility of CanAssist‐Breast is shown by comparison with the use of routine clinical methods such as, Ki‐67 and IHC4 score.

## 2 METHODS

### 2.1 Ethics approval

This study was approved by the institutional review board (IRB) and/or Ethics and Scientific Committees of participating hospitals. All studies were performed with the approval of the Bangalore Ethics Committee (ECR/87/Indt/KA/2013) and in accordance with the Declaration of Helsinki. The study was performed as per the committee recommendation. Patient information was anonymized prior to analysis.

### 2.2 Patients selection

We obtained postsurgical tumor samples in the form of formalin‐fixed paraffin‐embedded (FFPE) blocks from breast cancer patients. The treating hospital, from where the retrospective samples were obtained, provided all the patient and treatment follow‐up details such as age, year of diagnosis, type of surgery, tumor size and grade, hormone receptor status, node status, treatment regimen, date of recurrence, or last visit or death. All patients had hormone receptor‐positive disease. TNBC patients were excluded from the study. The patients were staged based on the AJCC 7th edition staging system. Patients with tumors with stage I (T1N0) and stage II (T1N1, T2N0, T2N1, and T3N0) were considered as early stage. Patients had undergone either mastectomy or breast‐conserving surgery or lumpectomy. Patients with a minimum of 5‐year follow‐up were included and this requirement was waived off only in patients with a recurrence at a distant site within the 5‐year period.

### 2.3 Tumor sample processing

Hematoxylin and eosin (H&E) staining was performed on the FFPE blocks to determine the percentage of tumor cells. Five consecutive sections taken further were used for IHC staining of the five biomarkers.

### 2.4 Immunohistochemistry

All the IHCs were performed at the OncoStem's CAP and ISO 15189 accredited central laboratory.

#### 2.4.1 CanAssist‐Breast test

The CAB test was performed as described earlier.^18^ IHC grading for membrane localization of CD44, ABCC4, and ABCC11, cytosolic localization of N‐ and Pan‐Cadherins along with three clinical parameters such as node status, tumor size, and Nottingham grade were used as inputs into SVM‐based algorithm that generates a risk score between 0 and 100. ROC (Receiver Operator Curve) analysis and determination of cutoff of 15.5 for low‐ and high‐risk categorization are described in the earlier work.^18^ The IHC staining protocol followed for CAB has been extensively validated for its analytical performance as per standard guidelines (*manuscript under review*).

#### 2.4.2 IHC4 score

IHC4 score was generated using a mathematical equation^12^ that uses the IHC gradings of ER, PR, Ki‐67, and HER2 and stratifies the patients for distant recurrence for a period of 5 years into three risk categories: low‐, intermediate‐, and high‐risk.^10^ ER/PR grading was performed as per ASCO and CAP guidelines^19^, ^20^ using a 1% cutoff for ER and PR positivity.

### 2.5 Study objective

The study objective was to evaluate effectiveness of CanAssist‐Breast in categorizing patients as low‐risk or high‐risk for distant recurrence within 5 years of diagnosis.

### 2.6 Statistical analyses

The following analyses were employed for assessing the contribution of the CanAssist‐Breast score in relation to clinical covariates: Kaplan‐Meier curves (GraphPad 8) and associated *P*‐values (Log‐rank test), hazard ratios (HR), and multivariate Cox proportional hazards model (MedCalc software). For multivariate analysis, the number of covariates was fixed at the initiation of the analysis and the method employed for multivariate analysis was “Entry.” Kaplan‐Meier survival curves and multivariate analysis were performed to calculate Distant Metastasis‐Free Survival (DMFS), recurrence rates, and HRs for CAB high‐risk vs low‐risk patients. *P*‐values were computed using log‐rank two‐sided test at 0.05 significance. DMFS is the time interval between the date of diagnosis of cancer and the last date of follow‐up in case of no event/recurrence with a minimum period of 5 years. TTP (time to tumor progression) is the time interval to develop the first distant recurrence from the date of diagnosis within 5 years.

### 2.7 Sample size estimation

Sample size and event rates were estimated using online tools (https://clincalc.com/stats/samplesize.aspx). The study parameters were derived from our pilot validation data published earlier.^18^ Following parameters were used to calculate the sample size: incidence rate of 7.5% in the low‐risk group, 26% in the high‐risk group, ratio between numbers of patients in high‐risk vs low‐risk groups at 0.48, alpha value of 0.05, and beta value of 0.1. With the statistical power of the study fixed at 90%, the minimum sample size was estimated as 182.

## 3 RESULTS

### 3.1 Description of validation cohort

Table 1 shows the details of the 857 sample cohort used in the clinical validation of CAB. It is a mixed cohort with 14% Caucasian and the remaining being South‐Asian patients. Of the total cohort, 61.85% of patients were aged above 50 years with the median age at disease presentation was 55 years. The cohort had a good representation of node‐negative (56.7%) and N1 (number of nodes with metastatic cells are 1‐3) (32.3%) patients. 65.3% of patients had stage II disease and 28% of patients had stage I disease. 82% of the patients expressed both ER and PR whereas 18% of the patients expressed ER alone and were negative for PR expression. All patients had a minimum follow‐up of 5 years (median‐5.5 years). 23% of patients were treated with endocrine therapy alone.

**Table 1:** Summary of the demographics and clinical features of the patient cohort that comprised CanAssist‐Breast (CAB) validation cohorts

Total cohort, n	857	
	Patients, n	% of patients
Age, years		
≤50	327	38.15
>50	530	61.85
Tumor size		
T1	240	28
T2	560	65.34
T3 + T4	57	6.65
Tumor Grade		
Well differentiated (Grade 1)	80	9.33
Moderately differentiated (Grade 2)	450	52.5
Poorly differentiated (Grade 3)	327	38.15
Number of nodes		
0 (N0)	486	56.7
1‐3 (N1)	277	32.32
4‐>10 (N2 + N3)	94	10.96
ER/PR status		
ER+/PR+	702	82
ER+/PR−	155	18
Treatment		
Endocrine therapy alone treated	195	22.8
Chemotherapy treated	662	77.2
Follow‐up		
Median	5.5 years	
Maximum	11.4 years	
Minimum	5 years	

ER, estrogen receptor; PR, progesterone receptor.

### 3.2 CAB test is prognostic in early‐stage breast cancer

#### 3.2.1 Risk stratification by CAB in the total cohort

**Figure 2:**
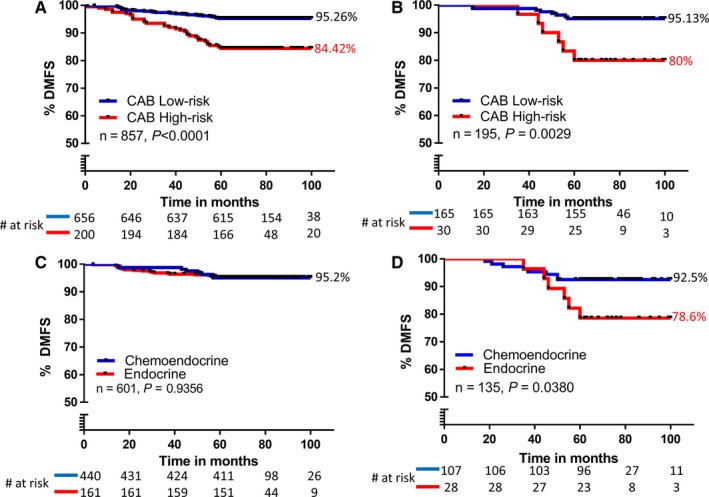
Risk classification by CanAssist‐Breast (CAB): Kaplan‐Meier plot of distant recurrence: stratified by CAB into low‐risk or high‐risk categories in the total validation cohort (n = 857) (A) subgroup of patients treated with endocrine therapy alone (n = 195) (B); endocrine therapy vs chemoendocrine therapy in CAB low‐risk category (C); endocrine therapy vs chemoendocrine in CAB high‐risk category (D)

The validation cohort of 857 samples was dichotomized into low‐risk or high‐risk categories based on CAB results. The categories were compared by Kaplan‐Meier survival analysis for DMFS for a period of 5 years (Figure 2A). There was a significant difference in the DMFS of patients defined as low‐risk (DMFS: 95%) or high‐risk (DMFS: 84%) by CAB in the total validation cohort (*P *<* *0.0001). The recurrence rate at 5 years was 3.5‐fold higher in patients categorized as high‐risk (15.58%, 95% CI: 7.098‐27.065) compared to those categorized as low‐risk (4.74%, 95% CI: 1.309‐11.716, *P *<* *0.0001) by CAB test (Figure 2A).

#### 3.2.2 Risk stratification by CAB in endocrine therapy alone cohort:

Risk categorization by CAB was also performed in 195 endocrine therapy alone treated cohort of patients. The analysis (Figure 2B) shows well separated survival curves for the low‐risk (DMFS: 95.16%) and high‐risk (DMFS: 80%) populations as predicted by CAB (*P *=* *0.002). The recurrence rate in the endocrine therapy alone cohort at 5 years was 20% (95% CI: 2.764‐48.585) in the high‐risk group compared to 4.84% (95% CI: 0.234‐21.993, *P *=* *0.0027) in the low‐risk group, indicating that the CAB high‐risk group had a fourfold higher recurrence rates compared to low‐risk category (Figure 2B).

#### 3.2.3 Improved DMFS in CAB high‐risk patients with chemotherapy

Next, we compared usefulness of CAB in stratifying endocrine or chemoendocrine (chemotherapy along with endocrine therapy) treated early‐stage breast cancer patients. As shown in Figure 2C, patients stratified as “low‐risk” by CAB had identical DMFS of 95.2% (*P *=* *0.935) independent of if they were treated with endocrine or chemoendocrine therapy (Figure 2C). On the contrary, in patients from CAB high‐risk category, those patients treated with chemotherapy (plus endocrine therapy) had about 13.9% improved DMFS of 92.5% as compared to patients who were treated with endocrine therapy alone (DMFS =78.6%) as shown in Figure 2D. This indicates that only patients stratified as “high‐risk” by CAB benefit from added chemotherapy to improve DMFS.

### 3.3 CAB can prognosticate in both node‐negative and node‐positive patients

**Figure 3:**
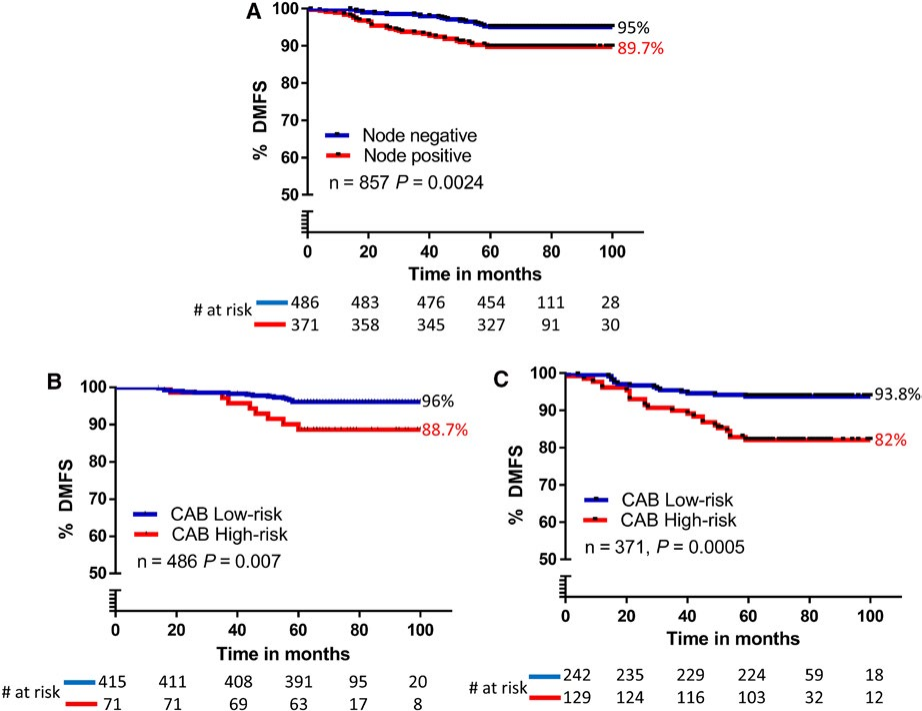
Kaplan‐Meier survival analysis of distant recurrence in the validation cohort by node status (A), low‐ and high‐risk groups by CanAssist‐Breast (CAB) in node‐negative (B), and node‐positive patients (C)

Node‐positive patients are generally perceived to have a higher risk for recurrence.^21^ Based on node status alone, we observed a 5.3% difference in DMFS (node‐negative DMFS: 95%; node‐positive DMFS: 89.7%) (Figure 3A) with *P *=* *0.0024. When the same cohort was stratified by CAB, the results showed a higher difference in DMFS (10.84%) between the low‐ and high‐risk categories with a highly significant *P*‐value of <0.0001 (Figure 2A) suggesting that CAB is a better prognostic predictor in comparison to node status. Next, we performed univariate and multivariate analysis on total cohort and found that in both analysis CAB risk score had a greater hazard ratio (HR) with higher significant *P*‐value than node status (Univariate: CAB risk score‐3.462, *P *<* *0.0001; node status‐2.153, *P *=* *0.0024, Supporting Information Table S1; multivariate: CAB risk score‐3.2048, *P < *0.0001, node status‐1.7258, *P *=* *0.0587, Table 2). We observed similar results when univariate and multivariate analysis were performed in endocrine therapy alone cohort (Univariate: CAB risk score‐4.363, *P *=* *0.0029, node status‐1.659, *P *=* *0.4322, Supporting Information Table S1; multivariate: CAB risk score‐4.1377, *P *=* *0.0118; node status‐1.11, *P *=* *0.8847, Table 2).

**Table 2:** Multivariate Cox Proportional Hazard Analysis for CAB risk score and other covariates in the total and endocrine therapy cohort. Survival coding in the analysis: patients aged below 50, 1 and above 50, 0; ER/PR: ≤20, 1 and >20, 0; treatment: endocrine, 1 and chemoendocrine, 0; tumor size: T2 + T3 + T4, 1 and T1, 0; node status: node positive, 1 and node negative, 0; grade: grade 3, 1 and grade 1 and 2, 0; CAB risk score: high‐risk score, 1 and low‐risk score, 0

Total cohort (n = 857)				Endocrine therapy cohort (n = 195)		
Covariate	HR	*P*‐value	95% CI	HR	*P*‐value	95% CI
Age	1.5670	0.0964	0.9228 to 2.6612	2.1746	0.2336	0.6057 to 7.8073
ER	1.1246	0.7445	0.5550 to 2.2789	0.6503	0.696	0.0751 to 5.6322
PR	1.6166	0.0672	0.9666 to 2.7037	1.2906	0.6698	0.3996 to 4.1687
Chemotherapy treatment	1.7448	0.1100	0.8815 to 3.4534	Not applicable		
Node status	1.7258	0.0587	0.9802 to 3.0387	1.11	0.8847	0.2706 to 4.5541
Tumor size	1.3307	0.3849	0.6985 to 2.5350	1.6951	0.3577	0.5505 to 5.2194
Grade	0.6481	0.1182	0.3761 to 1.1167	1.8976	0.2536	0.6319 to 5.6986
CAB risk score	3.2048	<0.0001	1.8849 to 5.4489	4.1377	0.0118	1.3691 to 12.5047

ER, estrogen receptor; PR, progesterone receptor; HR, hazard ratio; CI, confidence interval.

Studies have shown that 25%‐30% of node‐positive patients may be at low‐risk for recurrence with loco‐regional therapy and remain distant metastasis‐free even without adjuvant chemotherapy.^22^, ^23^ Thus, a significant proportion of node‐positive patients could avoid overtreatment with chemotherapy if accurately identified. At the same time, few node‐negative patients are benefitted by adjuvant chemotherapy resulting in reduced recurrence rates.^1^, ^24^ To evaluate if CAB would be able to identify these patients, we performed a subgroup analysis on cohorts of node‐negative (n = 486) and node‐positive (n = 371) patients independently using CAB (Figure 3B and C). The Kaplan‐Meier analysis showed that both node‐negative (Figure 3B, DMFS: 96% in low‐risk vs 88.7% in high‐risk, *P *=* *0.007) and node‐positive (Figure 3C, DMFS: 93.8% in low‐risk vs 82% in high‐risk, *P *=* *0.0005) patients were distinctly separated by CAB. There were significant proportions of patients in the low‐risk category in both node‐negative (85%) and node‐positive (65%) subgroups, albeit with a greater percentage in node‐negative subgroup. Comparison of recurrence rates across low‐ and high‐risk categories between node‐negative and node‐positive subgroups indicated that they were statistically not different from each other: CAB low‐risk, 4% in node‐negative vs 6% in node‐positive (*P *=* *0.2040, Figure 3B and C) and CAB high‐risk, 11.3% in node‐negative vs 18% in node‐positive (*P *=* *0.2129, Figure 3B and C). Moreover, CAB high‐risk patients had threefold higher recurrence rates compared to CAB low‐risk patients in both node‐positive and node‐negative subgroups. A similar analysis in the endocrine therapy alone treated cohort yielded a fivefold difference in recurrence rates between CAB risk categories (CAB low‐risk: 4%; CAB high‐risk: 20%, data not shown) in the node‐negative patients. This indicated that CAB was successful in identifying node‐negative patients (typically considered clinically low‐risk) who would have a better prognosis with chemoendocrine therapy.

### 3.4 CAB is an independent predictor of prognosis

We further tested whether CAB risk score is an independent indicator of distant recurrence in the total cohort and in endocrine therapy alone cohort using a multivariate Cox proportional hazards model. The analysis included standard prognostic factors such as age, ER/PR status, tumor size, node status, and grade along with CAB risk score (Table 2). In absolute terms, chemotherapy benefit rates in early breast cancer are known to be modest.^3^ However, since 77% patients (n = 662) in the validation cohort underwent chemoendocrine therapy, we included chemotherapy as a covariate in the multivariate analysis of the entire cohort to determine if chemotherapy benefit could confound the prognostic performance of CAB. Results in Table 2 show that neither chemotherapy nor any other parameter tested was a significant factor in determining prognosis in this cohort. The significance of CAB risk score is evident by higher HR with a highly significant *P*‐value (HR: 3.2048, 95% CI 1.8849‐5.4489, *P *<* *0.0001, Table 2) compared to all parameters tested.

In the endocrine therapy alone cohort as well, CAB risk score was the most significant predictor of prognosis (HR: 4.1377, 95% CI 1.3691‐12.5047, *P *=* *0.0118, Table 2) when compared to prognostic factors, age, and ER/PR status. The lower HR for the CAB risk score in the total cohort (3.2048) in comparison to the endocrine therapy treated alone cohort (4.1377) could be due to the modest benefit from chemotherapy, which is also evident from the higher recurrence rates in patients stratified as high‐risk by CAB (Figure 2A and B). Patients stratified as high‐risk for recurrence from the endocrine therapy alone cohort had a recurrence rate of 20% (Figure 2B) compared to a recurrence rate of 15.58% from the total cohort (Figure 2A).

### 3.5 Comparison of performance of CAB with Ki‐67 and IHC4 score

**Figure 4:**
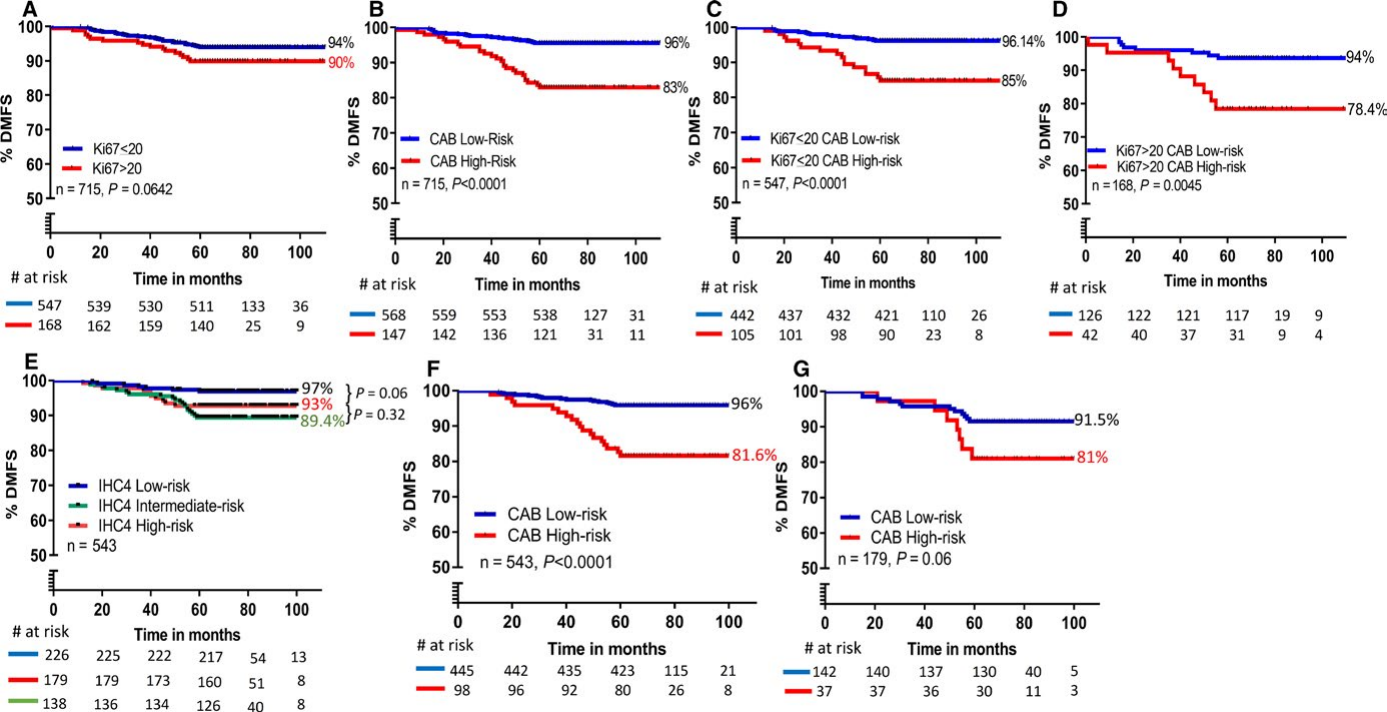
Comparison of CanAssist‐Breast (CAB) with Ki‐67 and IHC4 score: Kaplan‐Meier survival analysis of distant recurrence in the subset of validation cohort by Ki‐67 (n = 715) (A); CAB in the 715 cohort (B); CAB re‐stratification of low Ki‐67 patients (C); CAB re‐stratification of high Ki‐67 patients (D); IHC4 score (n = 543) (E); CAB in 543 cohort (F); CAB re‐stratification of IHC4 score intermediate‐risk category (n = 179) into low‐and high‐risk groups (G)

We compared the prognostic performance of CAB with Ki‐67, a routinely used prognostic and predictive biomarker using 20% staining as cutoff. Patients expressing high Ki‐67 are known to have bad prognosis with higher rates of recurrences.^25^, ^26^ In a subgroup of 715 patients, from the validation cohort, the difference in DMFS between patients expressing low (≤20) and high Ki‐67 (>20) was 4% with a nonsignificant *P*‐value of 0.0642 (Figure 4A). The data were similar when we used a 14% cutoff for Ki‐67 (data not shown). CAB stratified the same subgroup of patients (n = 715) into low‐ and high‐risk categories with a difference in DMFS of 13%, *P *<* *0.0001 (Figure 4B). This improved risk stratification of patients by CAB prompted us to re‐stratify these low and high Ki‐67 risk categories by CAB. CAB re‐stratified 19% of low Ki‐67 patients into high‐risk (Figure 4C) and 75% of high Ki‐67 patients into low‐risk category (Figure 4D) with significant *P*‐values (low Ki‐67 re‐stratification: *P *<* *0.0001; high Ki‐67 re‐stratification: *P *=* *0.0045; Figure 4C and D). It is worth noting that both re‐stratifications of low and high Ki‐67‐based risk groups by CAB yielded in risk categories which had a difference in DMFS of >11% (Figure 4C and D).

IHC4 score was performed on a subgroup of 543 patients from the validation set (Figure 4E). IHC4 score divided patients into three risk groups with 42% patients falling into low‐risk, 33% into the intermediate‐risk, and 25% into high‐risk categories. The low‐risk group (n = 226) had higher DMFS (96%) and was separated from the intermediate‐risk (n = 179) (DMFS: 89%, *P *=* *0.002) and high‐risk (n = 138) (DMFS: 91%, *P *=* *0.06) groups (Figure 4E). Both intermediate‐ and high‐risk groups showed >2.4‐fold higher rate of distant recurrence at 5 years compared to the low‐risk group (Figure 4E and Supporting Information Table S2). 95% confidence intervals and *P*‐values for these three IHC4 risk groups are provided in Supporting Information Table S2. Figure 4F shows risk stratification of the same cohort by CAB, where the low‐ and high‐risk patients were distinctly and significantly separated (*P *<* *0.0001) with a difference in DMFS of 15%. The DMFS in low‐risk groups of both tests was almost similar; (low‐risk category, DMFS: ~97% for IHC4 score and DMFS: 96% for CAB test) however, CAB (n = 445, 82%) stratified more patients into low‐risk as compared to IHC4 score (n = 226, 41.6%). Additionally, we assessed if CAB could further stratify the 179 patients (33%) categorized as intermediate‐risk by IHC4 score into low‐and high‐risk categories distinctly. As shown in Figure 4G, CAB was able to segregate 79% of the 179 patients categorized as intermediate‐risk by IHC4 score into discrete low‐risk group (n = 142, DMFS: 91%) and the remaining 21% as high‐risk group (n = 37, DMFS: 81%) (*P *=* *0.06).

## 4 DISCUSSION

Recently, we have described the development of CanAssist‐Breast.^18^ The pilot validation of CAB showed that it effectively segregated the patients into low‐ and high‐risk categories for distant recurrence in the first 5 years.^18^

Clinical parameters are reported to add additional value to the prognostic tests available. Prosigna^27^ and Oncotype DX^28^ showed an increased HR (high‐ vs low‐risk) and an increased percentage of patients in the low‐risk category with the addition of clinical parameters. EPClin,^29^ with clinical parameters added to the EndoPredict test, showed an increase in DMFS of low‐risk patients with an increased absolute risk reduction between the two risk groups. Therefore, we anticipated that addition of clinical parameters to our model will optimize the test performance. On the contrary, we observed that the clinical parameters alone were not sufficient in providing effective prognostication as clinical parameters, namely node status, tumor size, and grade did not have a higher significant HR compared to CAB risk score in the multivariate analysis.

The identical DMFS in the low‐risk category of both endocrine therapy alone (DMFS: 95.13%, Figure 2B) and the total cohort (DMFS: 95.26%, Figure 2A) suggests that chemotherapy did not affect DMFS in the low‐risk category although 77% of patients in the total cohort underwent chemoendocrine treatment. This finding is further substantiated by another comparative analysis, where we find identical DMFS in low‐risk categories of endocrine therapy cohort and chemoendocrine therapy cohort (DMFS: 95.2%, Figure 2C). However, higher DMFS (DMFS: 92.5%) in high‐risk patients of chemoendocrine cohort compared to that of endocrine therapy cohort (DMFS: 78.6%, Figure 2D), indicates the potential benefit of chemotherapy in the high‐risk patients. Nonetheless, the possible bias involved in the clinician's decision in not considering these patients for chemotherapy cannot be ruled out. Oncotype Dx could conclusively predict the benefit from adjuvant chemotherapy in high‐risk patients (recurrence score ≥31) within 5 years of diagnosis in node‐negative patients^30^ and node‐positive patients^31^ using two independent randomized prospective trials. Hence, to conclusively show the benefit of chemotherapy in high‐risk patients stratified by CAB a similar randomized clinical trial is necessary, a limitation of this current study and attempts are underway to conduct a randomized clinical trial.

CanAssist‐Breast identified 17% of node‐negative patients as high‐risk (Figure 3B). Only 15% of node‐negative patients with tumors >1 cm are at risk of recurrence within 5 years of diagnosis.^32^ NSABP 20 trial conducted in node‐negative patients showed that these patients have increased survival rates with chemotherapy compared to patients who were treated with endocrine therapy alone.^24^ Our data further show that a significant number of node‐positive patients (65%) are stratified by CAB as low‐risk. The recurrence rates in the CAB low‐risk patients in both node‐positive and node‐negative groups were very comparable and statistically nonsignificant. This suggests that all node‐positive patients do not benefit from chemotherapy and CAB can help segregate these patients effectively. Recommendations by St. Gallen's panel that node‐positive patients with 1‐3 nodes, with/without HER2 amplification, will respond to endocrine therapy alone^23^ supports our observation that not all node‐positive patients need chemotherapy. Similar recurrence rates (with a nonsignificant *P*‐value) in both low‐ and high‐risk categories by CAB in node‐negative and positive subgroups indicate that CAB could stratify patients independent of node status and significant proportions of node‐positive patients could be spared chemotherapy. Further meta‐analysis has shown that proportion of reduction in recurrence rates due to chemotherapy are similar in node‐negative and node‐positive patients^1^ in early‐stage breast cancer patients. This data along with HRs in multivariate (Table 2) and univariate (Supporting Information Table S1) analysis indicate that risk stratification based on CAB was more accurate. Since CAB assesses tumor biology with respect to tumor recurrence in depth, it is in line with the current understanding of the importance of biology over anatomy.^33^ We, therefore believe that it is important to consider multigene/biomarker tests that integrate prognostic features of tumor biology in addition to the conventional clinical indicators to treat patients effectively.

Risk stratification performed by CAB was more accurate compared to Ki‐67 and IHC4 score. In both low and high Ki‐67 subgroups, CAB was further able to substratify into distinct low‐ and high‐risk categories. CAB could re‐stratify the 33% IHC4 score based “intermediate‐risk” patients into low‐ and high‐risk categories, with a difference in DMFS of 9.5% providing more clinical actionability for this group of patients. Taken together, these results suggest the enhanced prognostic utility of CAB over IHC4 score and Ki‐67.

The data demonstrate the superior performance of CAB test over standard clinical parameters like node status, tumor stage, and Ki‐67 biomarker status. It also provided better clinical decision support than the IHC4 score. The validated CAB is thus an accurate and affordable prognostic test.

## 5 CONCLUSION

In summary, this manuscript describes the clinical validation of CanAssist‐Breast. We show here that CAB is robustly able to segregate early‐stage HR+ patients into low‐vs high‐risk categories for distant recurrence. The ability of CAB to segregate patients independent of node status coupled with it being the most significant predictor of cancer recurrence in a multivariate analysis (in comparison with various clinical and routinely used biomarkers) indicates the contribution of CAB biomarkers in predicting the risk of distant recurrence beyond routine parameters. The biomarkers chosen, essentially describe the biology of the tumor with relevance to recurrence, beyond proliferation. Going with the more recent trend of using tumor biology over anatomy, we believe CAB is a robust and useful tool in deciding treatment options for the intended use early‐stage breast cancer patients in low‐resource settings globally.

## AVAILABILITY OF DATA AND MATERIALS

The datasets generated during and/or analyzed during the current study are available from the corresponding author on reasonable request.

## CONSENT FOR PUBLICATION

Not applicable.

## CONFLICT OF INTEREST

All authors are employees/consultants at OncoStem Diagnostics Private Limited which has developed the CanAssist‐Breast test. MMB and CR are co‐inventors on a patent application related to this article. All other authors have no other competing interests to declare.

## ETHICS APPROVAL

Clearance for the samples used in this study has been obtained from the ethical committees of the respective hospitals.
