# Supplementary material for: Clinical validation of an immunohistochemistry‐based CanAssist‐Breast test for distant recurrence prediction in hormone receptor‐positive breast cancer patients
Source: Cancer Med. 2019 Mar 7;8(4):1755–64. doi: 10.1002/cam4.2049 (PMC6488210; doi:10.1002/cam4.2049)
Supplement: Supplementary file 1 [file CAM4-8-1755-s001.docx]

Supporting Information Table 1:

| **Total cohort (n=857)** | | | | **Endocrine therapy cohort (n=195)** | | |
| --- | --- | --- | --- | --- | --- | --- |
| **Covariate** | **HR** | ***p*-value** | **95% CI** | **HR** | ***p*-value** | **95% CI** |
| CAB risk score | 3.462 | <0.0001 | 2.697-8.9 | 4.363 | 0.0029 | 0.9926-19.18 |
| Node status | 2.153 | 0.0024 | 1.307-3.58 | 1.659 | 0.4322 | 0.3685-7.465 |

HR: hazard ratio, CI: confidence interval

Supporting Information Table 2:

| **IHC4 Risk category** | **Recurrence rates** | ***p*-value** | **95% CI** |
| --- | --- | --- | --- |
| Low-risk | 3.103 | 0.0112 | 0.068-19.1 |
| Intermediate-risk | 10.615 | 0.0112 | 2.96-23.923 |
| High-risk | 7.246 | 0.0112 | 0.76-24.365 |

CI: confidence Interval
